# Epigenetic Ageing and Breast Cancer Risk: A Systematic Review

**DOI:** 10.1002/cam4.70355

**Published:** 2024-11-11

**Authors:** Emily McLennan, Danmeng Lily Li, Melissa C. Southey, Pierre‐Antoine Dugué

**Affiliations:** ^1^ Precision Medicine, School of Clinical Sciences at Monash Health Monash University Clayton Victoria Australia; ^2^ Centre for Epidemiology and Biostatistics, Melbourne School of Population and Global Health The University of Melbourne Parkville Victoria Australia; ^3^ Cancer Epidemiology Division, Cancer Council Victoria Melbourne Victoria Australia; ^4^ Department of Clinical Pathology The University of Melbourne Parkville Victoria Australia

## Abstract

**Background:**

Age is one of the strongest risk factors for breast cancer. Measures of biological age based on DNA methylation have gained popularity for their strong association with risk of many diseases, including cancer, which may help to identify high‐risk subgroups for targeted prevention.

**Methods:**

We carried out a systematic review of prospective studies that examined the association of methylation‐based markers of ageing with risk of invasive breast cancer in healthy (breast cancer‐free) women, published up to May 2023. The search of three databases (MEDLINE, EMBASE and Web of Science) identified 2913 individual abstracts eligible for screening. Risk of bias assessment was conducted using ROBINS‐E.

**Results:**

Ten prospective studies met the eligibility criteria, and these were heterogeneous in design and findings. The most frequently assessed epigenetic ageing measures were Horvath's first‐generation clock, *PhenoAge* and *GrimAge*. Four studies reported mainly positive associations, five null associations and one reported a negative association. These associations were generally weak and the results were not consistent across epigenetic ageing measures.

**Conclusion:**

The summarised evidence is insufficient to support a role for current epigenetic ageing measures to stratify breast cancer risk.

**PROSPERO Registration:** This systematic review was registered in the International Prospective Register of Systematic Reviews (PROSPERO: CRD42023417559)

## 1 Introduction

Age is one of the strongest risk factors for developing breast cancer in women [1]. Beyond chronological age, ageing can also be defined in terms of biological age, that is, the physiological integrity of tissues, organs and cells [2], which results from the accumulation of environmental/lifestyle exposures over the life course, health conditions and genetic predisposition, and better reflects susceptibility to disease [3]. Of the hallmarks of ageing [4], epigenetic alterations have received considerable attention, which has prompted the development of measures of epigenetic ageing (‘epigenetic clocks’) to quantify the biological ageing of tissues, using DNA methylation data at dozens or hundreds of CpGs across the genome [5].

Despite extensive research and treatment progress, breast cancer remains one of the most commonly diagnosed cancer worldwide and a leading cause of cancer‐related death [6]. Reliable molecular markers that can identify high‐risk subgroups before they develop breast cancer may help reduce the burden of disease [7]. Exploration of the markers of ageing might provide insights into the link between ageing and breast cancer, as well as improvements to risk prediction and early detection. While strong associations between epigenetic ageing and risk of disease (including cancer) have been consistently reported in numerous contexts [3, 8], the findings for breast cancer appear to be inconsistent [9, 10, 11].

Current breast cancer screening programmes while effective at reducing mortality risk are mainly targeted at specific age population subgroups and generally lack the ability to take into consideration individualised risk variation based on molecular markers (with the exception of some subgroups such as carriers of *BRCA1* and *BRCA2* pathogenic mutations). This systematic review aimed to summarise the evidence on the association of epigenetic ageing markers with breast cancer risk and consider their potential value as a means to improve risk prediction.

## 2 Methods

This systematic review was registered with PROSPERO prior to commencing (Registration Number: CRD42023417559) and follows the Preferred Reporting Items for Systematic Reviews and Meta‐Analyses (PRISMA 2020) guideline (Appendix S1) [12].

### 2.1 Eligibility Criteria

Studies deemed eligible for inclusion were as follows: (i) peer‐reviewed articles with a full‐text available in English language and published prior to the search carried out on 3 May 2023; (ii) studies with a prospective design in women free of breast cancer at DNA methylation measurement; and (iii) studies with primary outcome breast cancer incidence, a breast cancer case being defined as a first diagnosis of invasive breast cancer of any molecular subtype or stage. We defined as ‘methylation‐based measures of ageing’: the five first‐generation Horvath and Hannum clocks and derivatives [13], *PhenoAge GrimAge*, and their principal component‐based derivatives [14, 15, 16], *DunedinPoAm* and *DunedinPACE* [17, 18]. No restriction to a specific sample type was applied, although most studies would have measured DNA methylation in blood due to the less invasive collection technique compared with, for example, breast tissue.

### 2.2 Search Strategy and Data Extraction

The databases MEDLINE, EMBASE and Web of Science were searched on 3 May 2023. The search combined the key terms epigenetic(s)/DNA methylation/methylation, age/ageing/ageing and breast cancer/breast neoplasm (Appendix S2). The reference lists and citations of the included eligible studies were also searched for additional studies meeting the eligibility criteria. Two independent reviewers (EM and PAD) screened records at the title and abstract stage and the full‐text screening stage to determine eligibility for inclusion. Conflicts arising at either stage were resolved by a discussion between the two reviewers (EM and PAD) to reach a consensus.

### 2.3 Data Extraction

EM extracted the data from the included studies and the table was reviewed by PAD and DLL. We recorded information on study design, study population characteristics (country and ancestry), age, number of participants and breast cancer cases, follow‐up time, epigenetic ageing measure used, sample type, statistical method, effect estimate (95% confidence interval, P‐value) and its relevant unit. As adjustments for confounding factors varied across studies, data were extracted for both the least and most comprehensively adjusted models where applicable. Missing data, such as standard deviations, were retrieved by contacting authors [3, 19, 20] and one missing confidence interval was calculated from the sample size and P‐value [21]. The original analysis plan specified carrying out a meta‐analysis, but this was not undertaken due to (1) limited available summary estimates expressed on the same scale and (2) high heterogeneity in the included studies in terms of sample size and follow‐up time.

### 2.4 Risk of Bias Assessment

The risk of bias was assessed using the Risk of Bias in Non‐Randomised Studies—of Exposures (ROBINS‐E) [22] tool involving justification for the risk of bias (low risk, some concerns or high risk) in each of the seven domains and an overall risk of bias for each included study. For the one included Mendelian randomisation study, we examined whether sensitivity analyses to the MR assumptions were conducted. Consideration was given to whether potential publication and selective nonreporting bias might have been present and impacted our conclusions [23, 24].

## 3 Results

### 3.1 Study Selection

**FIGURE 1:**
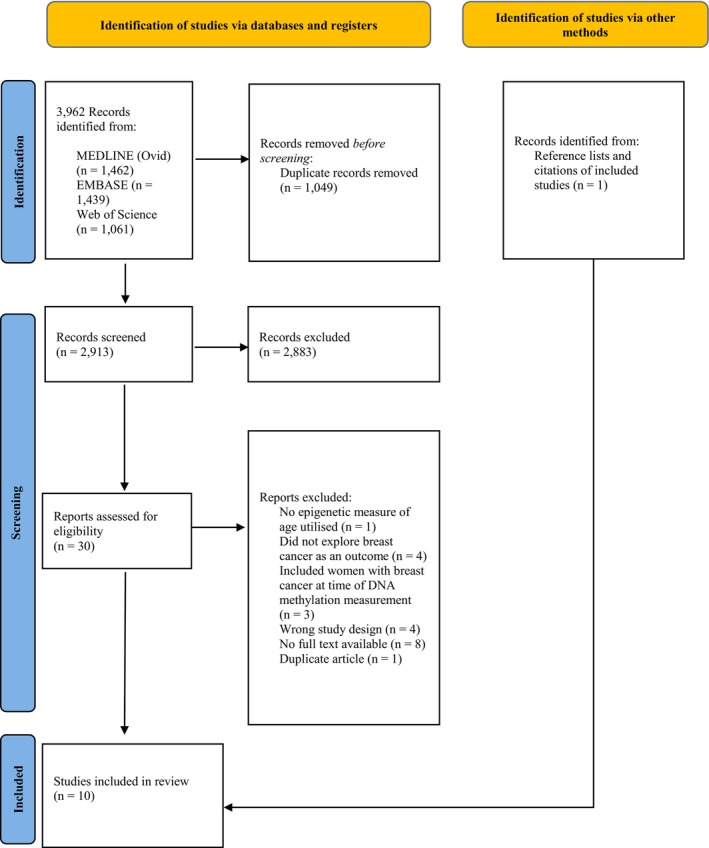
PRISMA flow diagram of the literature search and screening process [12].

Figure 1 outlines the PRISMA flow diagram of the study selection process. The database search returned 3962 records, and after duplicates (1049 records) were removed, the titles and abstracts of 2913 records were screened. Thirty full‐text articles were eligible for data extraction and nine full‐text articles were selected for data extraction [9, 10, 11, 19, 20, 21, 25, 26, 27]. One additional study [3] was identified from screening the reference list and citations of the included papers, making the total number of included studies equal to 10. For the 21 studies deemed ineligible for inclusion in the review at the full‐text screening stage, the reason for their exclusion is provided in Appendix S3.

### 3.2 Study Characteristics

Of the 10 included studies, there were four prospective case–control studies, two case–cohort studies, two cohort studies, one twin cohort study and a Mendelian randomisation study, with the longest follow‐up time being 17 years [19] (Table 1 **)**. The number of breast cancer cases ranged between 10 in the twin study [25] and 145,247 cases in the Mendelian randomisation study [27]. All studies were conducted in Western countries. Participant age at recruitment ranged between 23 [21] and 75 years [25]. Nine of the 10 studies [3, 9, 10, 11, 19, 20, 25, 26, 27] measured epigenetic ageing in blood samples and one in breast milk [21].

**TABLE 1:** Summary of study characteristics.

Study	Study design	Study population	Sample size (no. cases)	Epigenetic measure of age used	Sample type	Follow‐up time (years)	Effect estimates and analytical methods	Confounders
Durso, 2017 [26]	(Prospective) case–control study	Italy; EPIC‐Italy; Mean (SD) female controls; 52.57 (7.4) cases 52.37 (7.4)	845 (235)	Horvath, Hannum	Blood	~10 years	Wilcoxon–Mann–Whitney test.	—
Ambatipudi, 2017 [9]	Nested case–control study	France; EPIC cohort; Mean (SD) controls; 52.3 (8.94) cases; 52.3 (8.97)	902 (451)	Horvath	Blood	—	Odds ratios. Logistic regression	Model 1: unadjusted Model 2: alcohol consumption, full‐term pregnancy, BMI, education level, age at menarche and physical activity.
Kresovich, 2019a [10]	Case–cohort study	USA; the sister study (women with a family history of breast cancer); Mean age (SD) controls: 55.1 (9) cases: 57.7 (9)	2773 (1569)	GrimAge, Mortality Score (MS)	Blood	Mean follow‐up time 6 years	Hazard ratios. Cox proportional hazards model.	Model 1: unadjusted. Model 2: BMI, menopause, BMI–menopause interaction, physical activity, alcohol consumption, parity, age at first birth, age at menarche, breastfeeding duration and hormone therapy and oral contraception duration.
Kresovich, 2019b [20]	Case–cohort study	USA; the sister study (women with a family history of breast cancer); Mean age (SD) controls: 55.6 (9) cases: 58.1 (9)	2764 (1566)	Hannum, Horvath, PhenoAge	Blood	Mean follow‐up time 6 years	Hazard ratios. Cox proportional hazards model.	Model 1 (least adjusted): BMI, menopause, BMI–menopause interaction, alcohol consumption, parity, age at first live birth and age at menarche. Model 2 (most adjusted): BMI, menopause, BMI–menopause interaction, alcohol consumption, parity, age at first live birth, age at menarche and blood cell composition.
Hilary, 2020 [3]	Cohort study	United Kingdom; Generation Scotland; Mean age (SD) Discovery cohort: 51.4 (13.2) Replication cohort: 50.0 (12.5)	9307 (83)	Horvath, Hannum, PhenoAge, GrimAge, DunedinPoAm	Blood	Median time of onset from baseline 5.75 years range < 1 month to 13 years	Hazard ratios. Cox proportional hazards regression models.	Age
Salas, 2020 [21]	(Prospective) case–control study	USA; Umass and NHBCS; Mean age (range) controls; 33.2 (23–44) cases; 36.3 (29–45)	87 (26)	Horvath	Breast milk	Not specified	Mean difference. Linear mixed‐effects model	Unadjusted
Li, 2022 [37]	Cohort study (subset)	Germany; ESTHER; Mean (SD) Subset 1: 61.6 (6.5); Subset 2: 62.0 (6.7); Subset 3: 61.9 (6.4) Subset 4 Controls: 62.5 (6.4) Cases: 63.2 (6.0)	3052 (75)	PhenoAge, GrimAge, a revised	Blood	17 years	Hazard ratios. Subsets 1–3: Cox proportional hazard model; Subset 4: weighted Cox regression models	Model 1 (all four subsets): age, leukocyte composition and batch Model 2 (meta‐analysis of subsets 1, 2 and 3): age, leukocyte composition, batch, education level, smoking status, alcohol consumption, BMI, diabetes, menopausal status and postmenopausal hormone use
Morales Berstein, 2022 [27]	Mendelian randomisation	United Kingdom; BCAC, UK Biobank, FinnGen	549,075 (145,257)	Hannum, IEAA‐Horvath, PhenoAge, GrimAge	Blood	Not specified	Odds ratios. Main analysis: multiplicative random‐effects inverse variance‐weighted (IVW) MR. Pooled analysis: fixed‐effects meta‐analysis method	—
Bode, 2022 [25]	Twin cohort study	Finland; Finnish Twin Cohort Study; Mean age: 68.0 (range 58–75; SD 4.1)	46 twin pairs (10)	Horvath, IEAA‐ Horvath, Hannum, IEAA‐ Hannum, EEAA‐ Hannum, PhenoAge, GrimAge	Blood	8.4 (range 0.5–16.8)	Mean difference. Linear regression	Smoking status, matched for age and genetics (twin)
Dugué, 2022 [11]	Pooled analysis of four nested case–control studies	Australia; MCCS, EPIC‐Italy, EPIC‐IARC, PLCO; Median age of cases (IQR); MCCS 57.4 (49.8–62.8) EPIC‐IARC 53.4 (44.7–58.9) EPIC‐Italy 53.6 (48.0–57.6) PLCO 62.0 (58.3–66.2)	3532 (1655)	Horvath, IEAA‐Horvath, EEAA, Hannum, IEAA‐Hannum, PhenoAge, GrimAge	Blood	Time to diagnosis: median (IQR): MCCS 7.7 (4.4–11.1) EPIC‐IARC 7.7 (5.0–10.3) EPIC‐Italy 6.5 (2.5–10.6) PLCO 8.4 (5.6–10.5)	Odds ratios. Conditional or unconditional logistic regression; Pooled analysis: fixed‐effects meta‐analysis.	Model 1: unadjusted Model 2: Batch effects, white blood cell proportions, BMI, smoking, alcohol consumption and matched for age.

Abbreviations: BCAC = Breast Cancer Association Consortium, EPIC = European Prospective Investigation into Cancer and Nutrition, ESTHER = Epidemiological Study on the Chances of Cure, Early Detection and Optimised Therapy of Chronic Diseases in the Elderly Population, MCCS = Melbourne Collaborative Cohort Study, NHBCS = New Hampshire Birth Cohort Study, PLCO = Prostate, Lung, Colorectal and Ovarian Cancer Screening Trial, UMass = Molecular Biomarkers for Assessing Breast Cancer Risk Project at the University of Massachusetts Amherst.

### 3.3 Risk of Bias Assessment

The summary of the ROBINS‐E assessment is provided in Appendix S4. All nine observational studies were deemed to be of some concern overall [3, 9, 10, 11, 19, 20, 21, 25, 26]. Important confounding factors were identified to be age, adiposity, smoking, alcohol consumption, menopausal status and potentially white blood cell proportions, which were generally controlled for, at least partially. Risk of bias arising from measurement of the exposure was of some concern for all nine observational studies since they did not report on the reliability of the epigenetic ageing measures. For the remaining domains, there was low risk of bias in terms of selection of participants for the study, postexposure intervention, missing data, measurement of the outcome and selective reporting of results. Little risk of bias was concluded for the Mendelian randomisation study [27] as several sensitivity analyses were consistent with the main analysis.

### 3.4 Results of Individual Studies

#### 3.4.1 Case–Control Studies

A nested case–control study of 451 women with breast cancer and matched controls from EPIC‐IARC reported a positive association between intrinsic epigenetic age acceleration (IEAA) and breast cancer risk: adjusted OR per one unit IEAA: 1.04 (1.01–1.08), Table 2 [9]. This association was only apparent in postmenopausal women, OR = 1.07 (1.02–1.11). In a pooled analysis of women of White European ancestry (*n* = 1655 cases) from four nested case–control studies including most EPIC‐IARC cases [11], the estimates were consistent with a null association across the seven epigenetic ageing measures considered, for example, *PhenoAge* OR per SD: 1.01 (0.94–1.09) and *GrimAge* OR per SD: 1.03 (0.94–1.12). One small case–control study (*n* = 20 cases) that used breast milk found a 2.7‐year (95%CI: −0.2 to 4.4) higher *HorvathAge* in cases [21]. A larger prospective case–control study (EPIC‐Italy, *n* = 235 cases) provided only P‐values and boxplots for *HorvathAge* and *HannumAge*, showing no association [26].

**TABLE 2:** Effect estimates for the association between age‐adjusted epigenetic ageing and breast cancer risk.

Study	*N* women (*N* cases)	*HorvathAge* Est. (95% CI)	*HannumAge* Est. (95% CI)	*PhenoAge* Est. (95% CI)	*GrimAge* Est. (95% CI)	*DunedinPoAm* Est. (95% CI)	Overall association
Durso, 2017 [26]	845 (235)	No association					Null
Ambatipudi, 2017, [9] OR, per year	902 (451)						
Least adjusted		1.04 (1.01–1.08)					Positive
Most adjusted		1.04 (1.01–1.08)					
Kresovich, 2019a, [10] HR, per SD^a^	2773 (1569)						
Least adjusted					1.08 (0.99, 1.17)		Null
Most adjusted					1.04 (0.95, 1.14)		
Kresovich, 2019b, [20] HR, per 5 years^a^	2764 (1566)						
Least adjusted		1.09 (1.01, 1.18)	1.04 (0.95, 1.14)	1.13 (1.05, 1.22)			Positive
Most adjusted		1.10 (1.01, 1.20)	1.09 (0.99, 1.20)	1.17 (1.09, 1.27)			
Hillary, 2020, [3] HR, per SD^b^	9307 (83)	1.01 (0.79, 1.29)	1.24 (0.98, 1.57)	1.36 (1.07, 1.72)	1.19 (0.93, 1.53)	1.30 (1.01, 1.66)	Positive
Salas, 2020, [21] MD, years	87 (26)	2.7 (−0.2, 4.4)					Positive
Li, 2022, [37] HR, per SD^c^	3052 (75)						
Least adjusted				0.73 (0.56, 0.94)	0.67 (0.48, 0.93)		Negative
Most adjusted				0.65 (0.49, 0.86)	0.45 (0.25, 0.80)		
Morales‐Berstein, 2022, [27] OR, per year	549,075 (145,257)	0.99 (0.98, 1.00)	0.99 (0.97, 1.02)	0.99 (0.98, 1.01)	0.98 (0.95, 1.00)		Null
Bode, 2022, [25] MD, years	92 (10)	0.38 (−1.72, 2.49)	−1.92 (−5.21, 1.36)	1.11 (−3.72, 5.94)	−1.22 (−2.46, 0.02)		Null
Dugué, 2022, [11] OR, per SD^d^	3532 (1655)						
Least adjusted		1.00 (0.93, 1.07)	1.02 (0.95, 1.10)	1.01 (0.94, 1.09)	1.04 (0.97, 1.11)		Null
Most adjusted		1.02 (0.95, 1.10)	1.03 (0.95, 1.12)	1.01 (0.94, 1.09)	1.03 (0.94, 1.12)		

*Note:* When more than one model was presented in the corresponding study, the least and most adjusted models will both be presented.

Abbreviations: HR = hazard ratio, MD = Mean difference, OR = odds ratio.

^a^ In the Sister Study Kresovich et al. (2019) [10], [20], the SDs (age‐adjusted epigenetic age) were as follows (obtained from the corresponding author): *Hannum*: 4.6; *Horvath*: 4.4; *PhenoAge*: 5.8; *GrimAge*: 3.1.

^b^ In Hillary et al., (2020), the SD (age‐adjusted epigenetic age) were as follows (obtained from the corresponding author): *Hannum*: 3.5; *Horvath*: 4.3; PhenoAge: 4.6; *GrimAge*: 4.4; *DunedinPoAm*: 0.075.

^c^ In Li et al., (2022), the SDs were in previous publications with the same cohort [37]: *PhenoAge*: 5.7, *GrimAge*: 4.7.

^d^ In Dugué et al., (2022), the SDs were as follows (obtained from the corresponding author): *Hannum*: 11.8; *Horvath*: 9.5; *PhenoAge*: 9.8, *GrimAge*: 3.8.

#### 3.4.2 Case–Cohort Studies

Two studies used a case–cohort design (*n* > 1500 cases) within the sister study [10, 20]. One study found some evidence of an association for *GrimAge*: crude hazard ratio (HR) = 1.08 (0.99–1.17), adjusted HR = 1.04 (0.95–1.14) and the association appeared greater for postmenopausal women [20]. The other study reported positive associations for *HorvathAge*, *HannumAge* and *PhenoAge*, with adjusted HRs per 5 years ranging between 1.04 and 1.13, which appeared greater in white blood cell proportions adjusted models (1.09 to 1.17) [10], (Table 2 **)**.

#### 3.4.3 Cohort Studies

In the German prospective cohort study (*n* = 75 cases) [19], the authors found negative associations, *PhenoAge*, per SD, adjusted HR = 0.65 (0.49–0.86) and *GrimAge* HR = 0.45 (0.25–0.80). A Scottish prospective cohort study (83 cases) found markedly positive associations for *PhenoAge*: per SD, age‐adjusted HR = 1.36 (1.07–1.72) and *DunedinPoAm* HR = 1.30 (1.01–1.66) while the evidence was less clear for other epigenetic ageing measures: *GrimAge* HR = 1.19 (0.93–1.53), *HannumAge* HR = 1.24 (0.98–1.57) and *HorvathAge* HR = 1.01 (0.79–1.29) [3].

#### 3.4.4 Twin Studies

In the twin study, no other adjustment than that provided by the study design was made [25] and *GrimAge* appeared lower in women who developed breast cancer (mean difference *β* = −1.22 [−2.46 to −0.02]). Findings for other epigenetic ageing measures were consistent with a null association.

#### 3.4.5 Mendelian Randomisation Study

Genetically predicted levels of *GrimAge, PhenoAge*, *HannumAge* and *HorvathAge* were not found to be associated with breast cancer risk [27].

### 3.5 Selective Nonreporting and Publication Bias

Three of the larger studies reported no association [11, 20, 27], and some small studies reported positive and negative associations, suggesting that selective nonreporting bias was unlikely. In addition, two abstracts with no full‐text available reported positive and negative associations, respectively, suggesting low risk of publication bias.

## 4 Discussion

To the best of our knowledge, this is the first systematic review summarising the association of epigenetic ageing with breast cancer risk. The overall evidence across epigenetic ageing measures shows that any associations with breast cancer risk would be much weaker than for other cancer types [8] and diseases [3], and unlikely to provide improvements to risk prediction [28]. While this may be due in part to the limited number of studies to date, the more likely explanation is that environmental, lifestyle, clinical or other risk factors captured by the current epigenetic clocks do not play a strong role in breast cancer aetiology.

This systematic review was limited by the substantial heterogeneity in the included studies in terms of study design, sample size, follow‐up time and effect estimates. Another limitation was the relatively small number of eligible studies (*n* = 10). None of the reviewed studies assessed the repeatability of the epigenetic ageing measures; measurement error in biomarkers is nonnegligible [29] which usually biases risk estimates towards the null. Although participants were selected reasonably independently of the exposure and outcome, two of the studies followed women from the sister study [10, 20], which had a higher‐than‐average risk of breast cancer due to being selected as having a biological sister diagnosed with breast cancer. However, it is unclear through which mechanism such higher background risk might enhance the risk of breast cancer associated with epigenetic ageing. Most included studies adjusted for at least some of the predefined important confounding factors, although the list of adjustment variables varied across studies. Because biomarkers such as epigenetic ageing are typically reflective of the accumulation of environmental/lifestyle/hormonal exposures causing breast cancer, we also considered the unadjusted results, but these were similar, that is, not showing a substantial increase in breast cancer risk.

We did not restrict the exposure measurement to a particular sample type and the eligible studies identified were predominately based on blood samples. Breast tissue ageing might provide a more relevant assessment of breast cancer risk but sampling breast tissue is highly invasive and not feasible in large cohort studies. Previous studies have reported epigenetic ageing to be greater in healthy breast tissue than in blood collected from the same women at the same time point [30]. Besides, this review focused solely on risk of breast cancer but other outcomes such as progression and survival, which are important aspects for affected women, should be investigated in future studies. Our search strategy was very broad and while limited to studies in English language, we likely included all relevant studies. Two of the abstracts identified that appeared to meet the inclusion criteria had no associated full‐texts and we were not successful in retrieving more details about these studies. Interestingly, of those excluded abstracts, one reported an inverse association between epigenetic ageing and incident postmenopausal breast cancer risk [31]. Another abstract concluded a 9% increase in risk of breast cancer per *HorvathAge* ‘year’ [32]. There is no agreed definition and list of DNA methylation‐based measures of epigenetic ageing, and several studies have considered potentially valuable methylation‐based measures including *ELOVL2* methylation, an individual CpG that is strongly correlated with age [26], stochastic epigenetic mutations, which were reported to be associated with a fairly strong increase in breast cancer risk [33], methylation‐based telomere length, which showed weak or null association with cancer risk [3, 8], and Zhang's mortality risk score, which is widely studied but weakly correlated with age [34]. Methylation‐based markers of breast cancer risk factors such as BMI [11] as well as methylation at specific genes [35] have also been investigated and it is likely that combining several genetic and epigenetic risk factors will improve risk prediction [36].

To conclude, current epigenetic ageing measures are unlikely to translate into meaningful improvements to individual breast cancer risk prediction.

## Author Contributions

**Emily McLennan:** conceptualization (equal), data curation (equal), investigation (equal), methodology (equal), writing – original draft (equal). **Danmeng Lily Li:** data curation (equal), investigation (equal), writing – review and editing (equal). **Melissa C. Southey:** project administration (equal), resources (equal), supervision (equal), writing – review and editing (equal). **Pierre‐Antoine Dugué:** conceptualization (equal), formal analysis (equal), methodology (equal), project administration (equal), supervision (equal), writing – original draft (equal).

## Conflicts of Interest

The authors declare no conflicts of interest.

## Supporting information


Appendix S1.

## Data Availability

Data sharing is not applicable to this article as no new data were created or analysed in this study.

## References

[1] N. Fakhri , M. A. Chad , M. Lahkim , et al., “Risk Factors for Breast Cancer in Women: An Update Review,” Medical Oncology 39 (2022): 197. 36071255 10.1007/s12032-022-01804-x

[2] S. Horvath and K. Raj , “DNA Methylation‐Based Biomarkers and the Epigenetic Clock Theory of Ageing,” Nature Reviews. Genetics 19 (2018): 371–384. 10.1038/s41576-018-0004-3 29643443

[3] R. F. Hillary , A. J. Stevenson , D. L. McCartney , et al., “Epigenetic Measures of Ageing Predict the Prevalence and Incidence of Leading Causes of Death and Disease Burden,” Clinical Epigenetics 12 (2020): 115. 32736664 10.1186/s13148-020-00905-6 PMC7394682

[4] C. Lopez‐Otin , M. A. Blasco , L. Partridge , M. Serrano , and G. Kroemer , “The Hallmarks of Aging,” Cell 153 (2013): 1194–1217. 23746838 10.1016/j.cell.2013.05.039 PMC3836174

[5] L. Oblak , J. van der Zaag , A. T. Higgins‐Chen , M. E. Levine , and M. P. Boks , “A Systematic Review of Biological, Social and Environmental Factors Associated With Epigenetic Clock Acceleration,” Ageing Research Reviews 69 (2021): 101348, https://linkinghub.elsevier.com/retrieve/pii/S1568163721000957. 33930583 10.1016/j.arr.2021.101348

[6] F. Bray , J. Ferlay , I. Soerjomataram , R. L. Siegel , L. A. Torre , and A. Jemal , “Global Cancer Statistics 2018: GLOBOCAN Estimates of Incidence and Mortality Worldwide for 36 Cancers in 185 Countries,” CA: A Cancer Journal for Clinicians 68 (2018): 394–424. 30207593 10.3322/caac.21492

[7] M. Widschwendter , A. Jones , I. Evans , et al., “Epigenome‐Based Cancer Risk Prediction: Rationale, Opportunities and Challenges,” Nature Reviews. Clinical Oncology 15 (2018): 292–309. 10.1038/nrclinonc.2018.30 29485132

[8] P. A. Dugué , J. K. Bassett , E. M. Wong , et al., “Biological Aging Measures Based on Blood DNA Methylation and Risk of Cancer: A Prospective Study,” JNCI Cancer Spectrum 5 (2021): pkaa109. 33442664 10.1093/jncics/pkaa109 PMC7791618

[9] S. Ambatipudi , S. Horvath , F. Perrier , et al., “DNA Methylome Analysis Identifies Accelerated Epigenetic Ageing Associated With Postmenopausal Breast Cancer Susceptibility,” European Journal of Cancer 75 (2017): 299–307. 28259012 10.1016/j.ejca.2017.01.014 PMC5512160

[10] J. K. Kresovich , Z. Xu , K. M. O'Brien , C. R. Weinberg , D. P. Sandler , and J. A. Taylor , “Methylation‐Based Biological Age and Breast Cancer Risk,” Journal of the National Cancer Institute 111 (2019): 1051–1058. 30794318 10.1093/jnci/djz020 PMC6792078

[11] P. A. Dugué , C. Bodelon , F. F. Chung , et al., “Methylation‐Based Markers of Aging and Lifestyle‐Related Factors and Risk of Breast Cancer: A Pooled Analysis of Four Prospective Studies,” Breast Cancer Research 24 (2022): 59. 36068634 10.1186/s13058-022-01554-8 PMC9446544

[12] M. J. Page , D. Moher , P. M. Bossuyt , et al., “PRISMA 2020 Explanation and Elaboration: Updated Guidance and Exemplars for Reporting Systematic Reviews,” BMJ 372 (2021): n160. 33781993 10.1136/bmj.n160 PMC8005925

[13] B. H. Chen , R. E. Marioni , E. Colicino , et al., “DNA Methylation‐Based Measures of Biological Age: Meta‐Analysis Predicting Time to Death,” Aging (Albany NY) 8 (2016): 1844–1865. 27690265 10.18632/aging.101020 PMC5076441

[14] M. E. Levine , A. T. Lu , A. Quach , et al., “An Epigenetic Biomarker of Aging for Lifespan and Healthspan,” Aging (Albany NY) 10 (2018): 573–591. 29676998 10.18632/aging.101414 PMC5940111

[15] A. T. Lu , A. Quach , J. G. Wilson , et al., “DNA Methylation GrimAge Strongly Predicts Lifespan and Healthspan,” Aging (Albany NY) 11 (2019): 303–327. 30669119 10.18632/aging.101684 PMC6366976

[16] A. T. Higgins‐Chen , K. L. Thrush , Y. Wang , et al., “A Computational Solution for Bolstering Reliability of Epigenetic Clocks: Implications for Clinical Trials and Longitudinal Tracking,” Nature Aging 2 (2022): 644–661. 36277076 10.1038/s43587-022-00248-2 PMC9586209

[17] D. W. Belsky , A. Caspi , L. Arseneault , et al., “Quantification of the Pace of Biological Aging in Humans Through a Blood Test, the DunedinPoAm DNA Methylation Algorithm,” eLife 9 (2020): e54870. 32367804 10.7554/eLife.54870 PMC7282814

[18] D. W. Belsky , A. Caspi , D. L. Corcoran , et al., “DunedinPACE, a DNA Methylation Biomarker of the Pace of Aging,” eLife 11 (2022): e73420. 35029144 10.7554/eLife.73420 PMC8853656

[19] X. Li , B. Schöttker , B. Holleczek , and H. Brenner , “Associations of DNA Methylation Algorithms of Aging and Cancer Risk: Results From a Prospective Cohort Study,” eBioMedicine 81 (2022): 104083. 35636319 10.1016/j.ebiom.2022.104083 PMC9157462

[20] J. K. Kresovich , Z. Xu , K. M. O'Brien , C. R. Weinberg , D. P. Sandler , and J. A. Taylor , “Epigenetic Mortality Predictors and Incidence of Breast Cancer,” Aging (Albany NY) 11 (2019): 11975–11987. 31848323 10.18632/aging.102523 PMC6949084

[21] L. A. Salas , S. N. Lundgren , E. P. Browne , et al., “Prediagnostic Breast Milk DNA Methylation Alterations in Women Who Develop Breast Cancer,” Human Molecular Genetics 29 (2020): 662–673. 31943067 10.1093/hmg/ddz301 PMC7068171

[22] ROBINS‐E Development Group , J. Higgins , R. Morgan , et al., “Risk of Bias in Non‐Randomized Studies—Of Exposure (ROBINS‐E),” BMJ 355 (2023): i4919.

[23] M. J. Page , J. E. McKenzie , and J. P. Higgins , “Tools for Assessing Risk of Reporting Biases in Studies and Syntheses of Studies: A Systematic Review,” BMJ Open 8 (2018): e019703. 10.1136/bmjopen-2017-019703 PMC5857645 29540417

[24] M. J. Page , J. P. Higgins , and J. A. Sterne , “Assessing Risk of Bias due to Missing Results in a Synthesis,” Cochrane Handbook for Systematic Reviews of Interventions (2019): 349–374.

[25] H. F. Bode , A. Heikkinen , S. Lundgren , J. Kaprio , and M. Ollikainen , “Differences in DNA Methylation‐Based Age Prediction Within Twin Pairs Discordant for Cancer,” Twin Research and Human Genetics 25 (2022): 171–179. 36073160 10.1017/thg.2022.32

[26] D. F. Durso , M. G. Bacalini , C. Sala , et al., “Acceleration of leukocytes' Epigenetic Age as an Early Tumor and Sex‐Specific Marker of Breast and Colorectal Cancer,” Oncotarget 8 (2017): 23237–23245. 28423572 10.18632/oncotarget.15573 PMC5410300

[27] F. Morales Berstein , D. L. McCartney , A. T. Lu , et al., “Assessing the Causal Role of Epigenetic Clocks in the Development of Multiple Cancers: A Mendelian Randomization Study,” eLife 11 (2022): 11. 10.7554/eLife.75374 PMC9049976 35346416

[28] P. A. Dugué , C. Yu , A. M. Hodge , et al., “Methylation Scores for Smoking, Alcohol Consumption and Body Mass Index and Risk of Seven Types of Cancer,” International Journal of Cancer 153 (2023): 489–498. 36919377 10.1002/ijc.34513

[29] P.‐A. Dugué , D. R. English , R. J. MacInnis , et al., “Reliability of DNA Methylation Measures From Dried Blood Spots and Mononuclear Cells Using the HumanMethylation450k BeadArray,” Scientific Reports 6 (2016): 30317. 27457678 10.1038/srep30317 PMC4960587

[30] M. E. Sehl , J. E. Henry , A. M. Storniolo , P. A. Ganz , and S. Horvath , “DNA Methylation Age Is Elevated in Breast Tissue of Healthy Women,” Breast Cancer Research and Treatment 164 (2017): 209–219. 28364215 10.1007/s10549-017-4218-4 PMC5487725

[31] A. M. Binder , L. Tinker , R. Wallace , et al., “Abstract PS7‐28: Association Between Epigenetic Age Acceleration and Postmenopausal Breast Cancer Risk in the Women's Health Initiative,” Cancer Research 81 (2021): PS7‐28‐PS7.

[32] E. W. Hofstatter , M. Levine , C. Hatzis , and L. Pusztai , “Abstract P3‐05‐01: Age‐Related Methylation Signals of Breast Cancer Risk in Blood,” Cancer Research 79 (2019): P3‐05‐01.

[33] A. Gagliardi , P.‐A. Dugué , T. H. Nøst , et al., “Stochastic Epigenetic Mutations Are Associated With Risk of Breast Cancer, Lung Cancer, and Mature B‐Cell Neoplasms,” Cancer Epidemiology, Biomarkers & Prevention 29 (2020): 2026–2037. 10.1158/1055-9965.EPI-20-0451 32788174

[34] X. Gao , E. Colicino , J. Shen , et al., “Comparative Validation of an Epigenetic Mortality Risk Score With Three Aging Biomarkers for Predicting Mortality Risks Among Older Adult Males,” International Journal of Epidemiology 48 (2019): 1958–1971. 31038702 10.1093/ije/dyz082 PMC6929530

[35] Z. Xu , D. P. Sandler , and J. A. Taylor , “Blood DNA Methylation and Breast Cancer: A Prospective Case‐Cohort Analysis in the Sister Study,” Journal of the National Cancer Institute 112 (2020): 87–94. 30989176 10.1093/jnci/djz065 PMC7489106

[36] J. K. Kresovich , Z. Xu , K. M. O'Brien , et al., “Blood DNA Methylation Profiles Improve Breast Cancer Prediction,” Molecular Oncology 16 (2022): 42–53. 34411412 10.1002/1878-0261.13087 PMC8732352

[37] X. Li , Y. Zhang , X. Gào , B. Holleczek , B. Schöttker , and H. Brenner , “Comparative Validation of Three DNA Methylation Algorithms of Ageing and a Frailty Index in Relation to Mortality: Results From the ESTHER Cohort Study,” eBioMedicine 74 (2021): 74. 10.1016/j.ebiom.2021.103686 PMC8609015 34808433

