# Supplementary material for: Epigenetic Ageing and Breast Cancer Risk: A Systematic Review
Source: Cancer Med. 2024 Nov 11;13(21):e70355. doi: 10.1002/cam4.70355 (PMC11555139; doi:10.1002/cam4.70355)
Supplement: Supplementary file 1 — Appendix S1. [file CAM4-13-e70355-s001.docx]

Appendix 1 PRISMA 2020 checklist.

| Section and Topic | Item # | Checklist item | Location where item is reported |
| --- | --- | --- | --- |
| **Title** | | |  |
| Title | 1 | Identify the report as a systematic review. | 1 |
| **Abstract** | | |  |
| Abstract | 2 | See the PRISMA 2020 for Abstracts checklist. | 2 |
| **Introduction** | | |  |
| Rationale | 3 | Describe the rationale for the review in the context of existing knowledge. | 3 |
| Objectives | 4 | Provide an explicit statement of the objective(s) or question(s) the review addresses. | 3 |
| **Methods** | | |  |
| Eligibility criteria | 5 | Specify the inclusion and exclusion criteria for the review and how studies were grouped for the syntheses. | 4 |
| Information sources | 6 | Specify all databases, registers, websites, organisations, reference lists and other sources searched or consulted to identify studies. Specify the date when each source was last searched or consulted. | 4 |
| Search strategy | 7 | Present the full search strategies for all databases, registers and websites, including any filters and limits used. | 4 |
| Selection process | 8 | Specify the methods used to decide whether a study met the inclusion criteria of the review, including how many reviewers screened each record and each report retrieved, whether they worked independently and if applicable, details of automation tools used in the process. | 4–5 |
| Data collection process | 9 | Specify the methods used to collect data from reports, including how many reviewers collected data from each report, whether they worked independently, any processes for obtaining or confirming data from study investigators and if applicable, details of automation tools used in the process. | 4–5 |
| Data items | 10a | List and define all outcomes for which data were sought. Specify whether all results that were compatible with each outcome domain in each study were sought (e.g., for all measures, time points and analyses), and if not, the methods used to decide which results to collect. | 4–5 |
|  | 10b | List and define all other variables for which data were sought (e.g., participant and intervention characteristics and funding sources). Describe any assumptions made about any missing or unclear information. | 4–5 |
| Study risk of bias assessment | 11 | Specify the methods used to assess risk of bias in the included studies, including details of the tool(s) used, how many reviewers assessed each study and whether they worked independently, and if applicable, details of automation tools used in the process. | 5 |
| Effect measures | 12 | Specify for each outcome the effect measure(s) (e.g., risk ratio and mean difference) used in the synthesis or presentation of results. | 4–5 |
| Synthesis methods | 13a | Describe the processes used to decide which studies were eligible for each synthesis (e.g., tabulating the study intervention characteristics and comparing against the planned groups for each synthesis (Item #5)). | 4–5 |
|  | 13b | Describe any methods required to prepare the data for presentation or synthesis, such as handling of missing summary statistics, or data conversions. | 4–5 |
|  | 13c | Describe any methods used to tabulate or visually display results of individual studies and syntheses. | 4–5 |
|  | 13d | Describe any methods used to synthesise results and provide a rationale for the choice(s). If meta-analysis was performed, describe the model(s) and method(s) to identify the presence and extent of statistical heterogeneity and software package(s) used. | 4–5 |
|  | 13e | Describe any methods used to explore possible causes of heterogeneity among study results (e.g., subgroup analysis and meta-regression). | N/A |
|  | 13f | Describe any sensitivity analyses conducted to assess robustness of the synthesised results. | N/A |
| Reporting bias assessment | 14 | Describe any methods used to assess risk of bias due to missing results in a synthesis (arising from reporting biases). | 5 |
| Certainty assessment | 15 | Describe any methods used to assess certainty (or confidence) in the body of evidence for an outcome. | Appendix 5 |
| **Results** | | |  |
| Study selection | 16a | Describe the results of the search and selection process, from the number of records identified in the search to the number of studies included in the review, ideally using a flow diagram. | 5 |
|  | 16b | Cite studies that might appear to meet the inclusion criteria, but which were excluded, and explain why they were excluded. | 5 |
| Study characteristics | 17 | Cite each included study and present its characteristics. | 5–6 |
| Risk of bias in studies | 18 | Present assessments of risk of bias for each included study. | 6 |
| Results of individual studies | 19 | For all outcomes, present, for each study: (a) summary statistics for each group (where appropriate) and (b) an effect estimate and its precision (e.g., confidence/credible interval), ideally using structured tables or plots. | 6–7 |
| Results of syntheses | 20a | For each synthesis, briefly summarise the characteristics and risk of bias among contributing studies. | N/A |
|  | 20b | Present results of all statistical syntheses conducted. If meta-analysis was done, present for each the summary estimate and its precision (e.g., confidence/credible interval) and measures of statistical heterogeneity. If comparing groups, describe the direction of the effect. | N/A |
|  | 20c | Present results of all investigations of possible causes of heterogeneity among study results. | N/A |
|  | 20d | Present results of all sensitivity analyses conducted to assess the robustness of the synthesised results. | N/A |
| Reporting biases | 21 | Present assessments of risk of bias due to missing results (arising from reporting biases) for each synthesis assessed. | 7 |
| Certainty of evidence | 22 | Present assessments of certainty (or confidence) in the body of evidence for each outcome assessed. | Appendix 5 |
| **Discussion** | | |  |
| Discussion | 23a | Provide a general interpretation of the results in the context of other evidence. | 7–8 |
|  | 23b | Discuss any limitations of the evidence included in the review. | 8 |
|  | 23c | Discuss any limitations of the review processes used. | 8 |
|  | 23d | Discuss implications of the results for practice, policy and future research. | 8–9 |
| **Other information** | | |  |
| Registration and protocol | 24a | Provide registration information for the review, including register name and registration number, or state that the review was not registered. | 3 |
|  | 24b | Indicate where the review protocol can be accessed, or state that a protocol was not prepared. | N/A |
|  | 24c | Describe and explain any amendments to information provided at registration or in the protocol. | 3 |
| Support | 25 | Describe sources of financial or nonfinancial support for the review, and the role of the funders or sponsors in the review. | N/A |
| Competing interests | 26 | Declare any competing interests of review authors. | N/A |
| Availability of data, code and other materials | 27 | Report which of the following are publicly available and where they can be found: template data collection forms; data extracted from included studies; data used for all analyses; analytic code; any other materials used in the review. | N/A |

Appendix 2 Search strategy.

**MEDLINE (Ovid) (1462 results).**

| # | Searches |
| --- | --- |
| 1 | epigenetic* |
| 2 | Methylation |
| 3 | DNA methylation/ |
| 4 | 1 or 2 or 3 |
| 5 | ageing or ageing |
| 6 | Age |
| 7 | exp Ageing/ |
| 8 | 5 or 6 or 7 |
| 9 | Breast |
| 10 | cancer* |
| 11 | 9 and 10 |
| 12 | neoplasm* |
| 13 | 9 and 12 |
| 14 | 11 or 13 |
| 15 | 4 and 8 and 14 |

**EMBASE (1439 results)**

| # | Searches |
| --- | --- |
| 1 | epigenetic* |
| 2 | Methylation |
| 3 | DNA methylation/ |
| 4 | 1 or 2 or 3 |
| 5 | ageing or ageing |
| 6 | Age |
| 7 | exp Ageing/ |
| 8 | 5 or 6 or 7 |
| 9 | Breast |
| 10 | cancer* |
| 11 | 9 and 10 |
| 12 | neoplasm* |
| 13 | 9 and 12 |
| 14 | 11 or 13 |
| 15 | 4 and 8 and 14 |

**Web of Science (1061 results)**

((TS = (epigenetic* OR methylation OR DNA methylation)) AND TS = (age OR ageing OR ageing)) AND TS = (breast AND cancer* OR breast AND neoplasm*))

Appendix 3 Studies excluded at full-text screening stage with the reason for exclusion.

| Studies | Reason for exclusion |
| --- | --- |
| Guan Z, Raut JR, Weigl K, Schöttker B, Holleczek B, Zhang Y, et al. Individual and joint performance of DNA methylation profiles, genetic risk score and environmental risk scores for predicting breast cancer risk. Mol Oncol. 2020;14:42–53. | No epigenetic measure of ageing utilised. |
| Miyano M, Shalabi S, Sayaman RW, Stampfer M, Seewaldt VE, LaBarge MA. Abstract 5682: Accelerated biological age is a driver of cancer susceptibility in genetic high risk breast tissue. Cancer Research. 2022;82:5682-. | No full-text article available. |
| Daw J, Saunders D, Vasquez A, Valencia C. Abstract B013: Epigenetic clocks and breast cancer outcomes: A scoping review. [R]. Cancer Research. 2023;83:B013-B. | No full-text article available. |
| Barrett JE, Herzog C, Kim Y-N, Bartlett TE, Jones A, Evans I, et al. Susceptibility to hormone-mediated cancer is reflected by different tick rates of the epithelial and general epigenetic clock. Genome Biology. 2022;23:52. | Included women with breast cancer at time of DNA methylation measurement—wrong population group. |
| Hofstatter EW, Horvath S, Chagpar AB, Wali V, Bossuyt V, Storniolo AM, et al. Comparison of epigenetic ageing in normal breast tissue from women with and without breast cancer. Journal of Clinical Oncology. 2017;35:1522-. | No full-text article available. |
| Sehl ME, Henry JE, Storniolo AM, Horvath S, Ganz PA. The Effects of Lifetime Oestrogen Exposure on Breast Epigenetic Age. Cancer Epidemiol Biomarkers Prev. 2021;30:1241–9. | Did not explore breast cancer as an outcome. |
| Valencia CI, Saunders D, Daw J, Vasquez A. DNA methylation accelerated age as captured by epigenetic clocks influences breast cancer risk. Front Oncol. 2023;13:1150731. | A scoping review with qualitative synthesis—wrong study design. |
| Chen M, Wong EM, Nguyen TL, Dite GS, Stone J, Dugué PA, et al. DNA methylation-based biological age, genome-wide average DNA methylation and conventional breast cancer risk factors. Sci Rep. 2019;9:15055. | Did not explore breast cancer as an outcome. |
| Rozenblit M, Hofstatter E, Liu Z, O'Meara T, Storniolo AM, Dalela D, et al. Evidence of accelerated epigenetic ageing of breast tissues in patients with breast cancer is driven by CpGs associated with polycomb-related genes. Clinical Epigenetics. 2022;14:30. | Included women with breast cancer at time of DNA methylation measurement—wrong population group. |
| Binder AM, Tinker L, Wallace R, Manson JE, Qi L, Bhatti P, et al. Abstract PS7-28: Association between epigenetic age acceleration and postmenopausal breast cancer risk in the Women's Health Initiative. Cancer Research. 2021;81:PS7-28-PS7-. | No full-text article available. |
| Hofstatter E, Levine M, Liu Z, O'Meara T, Dalela D, Pusztai L. Abstract P2-09-02: Evidence of accelerated epigenetic ageing of breast tissues in patients with breast cancer compared to women without cancer. Cancer Research. 2020;80:P2-09-2-P2--2. | No full-text article available. |
| Langevin SM, Pinney SM, Leung YK, Ho SM. Does epigenetic drift contribute to age-related increases in breast cancer risk? Epigenomics. 2014;6:367–9. | An editorial report—wrong study design. |
| Panjarian S, Madzo J, Keith K, Slater CM, Sapienza C, Jelinek J, et al. Accelerated ageing in normal breast tissue of women with breast cancer. Breast Cancer Research. 2021;23:58. | Included women with breast cancer at time of DNA methylation measurement—wrong population group. |
| Dugué PA, Bassett JK, Joo JE, Jung CH, Ming Wong E, Moreno-Betancur M, et al. DNA methylation-based biological ageing and cancer risk and survival: Pooled analysis of seven prospective studies. Int J Cancer. 2018;142:1611–9. | Did not explore breast cancer as an outcome. |
| Pierce BL. The ageing epigenome. eLife. 2022;11:e78693. | A review of recent literature—wrong study design. |
| Sehl ME, Guo W, Farrell C, Marino N, Henry JE, Storniolo AM, et al. Abstract P2-11-06: Differential gene expression patterns in healthy breast tissue exhibiting epigenetic age acceleration. Cancer Research. 2022;82:P2-11-06-P2-11-06. | No full-text article available. |
| Sehl ME, Henry JE, Storniolo AM, Ganz PA, Horvath S. DNA methylation age is elevated in breast tissue of healthy women. Breast Cancer Res Treat. 2017;164:209–19. | Did not explore breast cancer as an outcome. |
| Abstracts from the 19th International Congress on Twin Studies, 11–14 November 2021. Twin Research and Human Genetics. 2021;24:385–407. | Multiple abstracts from International Congress on Twin Studies—wrong study design |
| Castle JR, Lin N, Liu J, Wang C, Liu Y, He C. Abstract 827: Estimating breast tissue-specific epigenetic age using next-generation methylation sequencing data. Cancer Research. 2019;79:827-. | No full-text article available. |
| Hofstatter EW, Levine M, Hatzis C, Pusztai L. Abstract P3-05-01: Age-related methylation signals of breast cancer risk in blood. Cancer Research. 2019;79:P3-05-1-P3--1. | No full-text article available. |
| Morales-Berstein F, McCartney DL, Lu AT, Tsilidis KK, Bouras E, Haycock P, et al. Assessing the causal role of epigenetic clocks in the development of multiple cancers: a Mendelian randomisation study. medRxiv. 2021:2021.11.29.21266984. | Duplicate article |

Appendix 4 Risk of Bias in Non-Randomised Studies–of Exposures (ROBINS-E).

| Study | Domain 1: Risk of bias due to confounding | Domain 2: Risk of bias arising from measurement of the exposure | Domain 3: Risk of bias in selection of participants into the study (or into the analysis) | Domain 4: Risk of bias due to postexposure interventions | Domain 5: Risk of bias due to missing data | Domain 6: Risk of bias arising from measurement of the outcome | Domain 7: Risk of bias in selection of reported result | Overall risk of bias |
| --- | --- | --- | --- | --- | --- | --- | --- | --- |
| **Durso, 2017** | Some Concerns | Some Concerns | Low Risk of Bias | Low Risk of Bias | Low Risk of Bias | Low Risk of Bias | Low Risk of Bias | Some Concerns |
| **Ambatipudi, 2017** | Low Risk of Bias except for concerns about residual confounding | Some Concerns | Low Risk of Bias | Low Risk of Bias | Low Risk of Bias | Low Risk of Bias | Low Risk of Bias | Some Concerns |
| **Kresovich, 2019a** | Low Risk of Bias except for concerns about residual confounding | Some Concerns | Low Risk of Bias | Low Risk of Bias | Low Risk of Bias | Low Risk of Bias | Low Risk of Bias | Some Concerns |
| **Kresovich, 2019b** | Low Risk of Bias except for concerns about residual confounding | Some Concerns | Low Risk of Bias | Low Risk of Bias | Low Risk of Bias | Low Risk of Bias | Low Risk of Bias | Some Concerns |
| **Hilary,2020** | Some Concerns | Some Concerns | Low Risk of Bias | Low Risk of Bias | Low Risk of Bias | Low Risk of Bias | Low Risk of Bias | Some Concerns |
| **Salas, 2020** | Some Concerns | Some Concerns | Low Risk of Bias | Low Risk of Bias | Low Risk of Bias | Low Risk of Bias | Low Risk of Bias | Some Concerns |
| **Li, 2022** | Low Risk of Bias except for concerns about residual confounding | Some Concerns | Low Risk of Bias | Low Risk of Bias | Low Risk of Bias | Low Risk of Bias | Low Risk of Bias | Some Concerns |
| **Bode, 2022** | Low Risk of Bias except for concerns about residual confounding | Some Concerns | Low Risk of Bias | Low Risk of Bias | Low Risk of Bias | Low Risk of Bias | Low Risk of Bias | Some Concerns |
| **Dugué, 2022** | Low Risk of Bias except for concerns about residual confounding | Some Concerns | Low Risk of Bias | Low Risk of Bias | Low Risk of Bias | Low Risk of Bias | Low Risk of Bias | Some Concerns |
